# Comparative analysis of Mycoplasma Pneumoniae and Drug-induced Stevens-Johnson Syndrome/Toxic Epidermal Necrolysis in Children

**DOI:** 10.12669/pjms.41.3.10286

**Published:** 2025-03

**Authors:** Xiaoyuan Wu, Lei Kang, Yanhong Jia, Li Jia, Fang Guo

**Affiliations:** 1 Xiaoyuan Wu Department of Infectious Diseases, Hebei Children’s Hospital, 133 Jianhua South Street, Shijiazhuang 050000, Hebei Province, China; 2 Lei Kang Department of Pediatric Intensive Care Unit, Hebei Children’s Hospital, 133 Jianhua South Street, Shijiazhuang 050000, Hebei Province, China; 3 Yanhong Jia Department of Infectious Diseases, Hebei Children’s Hospital, 133 Jianhua South Street, Shijiazhuang 050000, Hebei Province, China; 4 Li Jia Department of Infectious Diseases, Hebei Children’s Hospital, 133 Jianhua South Street, Shijiazhuang 050000, Hebei Province, China; 5 Fang Guo Department of Infectious Diseases, Hebei Children’s Hospital, 133 Jianhua South Street, Shijiazhuang 050000, Hebei Province, China

**Keywords:** Children, Mcoplasma pnemanime, Stevens-Johnson syndrome, Toxic epidermal necrolysis

## Abstract

**Objective:**

To compare the differences between clinical characteristics, therapeutic management, and prognosis of mycoplasma pneumonia (MP) and drug-induced Stevens-Johnson syndrome (SJS)/toxic epidermal necrolysis (TEN) in children.

**Methods:**

This was a retrospective study. The clinical data of SJS and TEN patients admitted to Hebei Children’s Hospital from 2014–2024 were retrospectively analyzed and divided into the MP group and the drug group based on laboratory findings and the ALDEN algorithm for comparative study.

**Results:**

A total of 42 cases were included in the study. Among them, 20 cases were in the MP group and 22 cases were in the drug group. The median age of MP group was 108.0 (54.0, 129.0) months, which was greater than drug group with 42.0 (22.5, 75.0) months, and the difference was statistically significant (P < 0.05). Ten cases (50.0%) in the MP group had chest CT suggestive of consolidation of lung/pleural effusion, which was higher than the two cases (9.1%) in the drug group, with a statistically significant difference (P<0.05). Both groups were given systemic corticosteroids (Cs) treatment, and the proportion of children receiving Cs shock therapy combined with intravenous immunoglobulin (IVIG) in the MP group was significantly lower than that in the drug group, with a statistically significant difference (P<0.05). When SCORTEN score ≧3, the proportion of the MP group receiving Cs shock therapy and IVIG application increased. The median SCORTEN score in both groups was two, corresponding to a predicted mortality rate of 12.2%, whereas all children in the MP group survived and three died in the drug group, with an actual mortality rate of 13.6%.

**Conclusion:**

MP infection and drugs constitute the predominant triggers of SJS/TEN in children, with non-steroidal anti-inflammatory drugs (NSAIDs) and Chinese patent medicines (CPMs) as the main sensitizing drugs. For those with SCORTEN≦2 points, macrolides combined with conventional dose Cs may be the first-line treatment option for them.

## INTRODUCTION

Stevens-Johnson syndrome(SJS) and toxic epidermal necrolysis(TEN) are distinct stages of the same disease with immune-mediated involvement of the skin and mucous membranes, resulting in damage to organs such as the eyes, liver, kidneys, and lungs, with a morbidity and mortality rate ranging from 5.4% to 15.3%.^1^ Drugs and infections account for the induction of SJS/TEN.^2^ In recent years, mycoplasma pneumoniae (MP) has not only emerged as a major cause of respiratory infections in children^3^, but also induces MP-related extra pulmonary diseases (MpEPDS).^4^ Skin mucosal manifestations, including simple mucositis, urticaria, erythema multiforme, and extensive epidermal detachment, have been reported in 20% to 30% of MP infection cases.^5^ In 2015, Canavan et al.^6^ summarized the characteristics of the MP-related SJS population and proposed the term Mycoplasma-Induced Rash and Mucositis (MIRM). Case reports related to MIRM have gradually increased in recent years^7^, but there is a lack of pediatric cohort studies, and this criterion ignores the MP-induced TEN populations. In this study, a comparative analysis of MP and drug-induced SJS and TEN was performed by retrospectively analyzing the clinical characteristics, therapeutic management, and prognosis of the children.

## METHODS

This was a retrospective study. A total of 67 children with SJS, SJS/TEN overlap and TEN admitted to Hebei Children’s Hospital from January 2014 to January 2024 were retrospectively collected, of which 14 cases due to other well-defined etiologic agents, such as respiratory syncytial virus, novel coronavirus, influenza virus, coxsackievirus, herpesvirus, as well as 11 cases with unspecified aetiology were excluded, and 42 cases were ultimately included in the study. The included patients were categorized into 20 and 22 cases in the MP group and the drug group, respectively. Presence of evidence of MP infection based on clinical or laboratory findings (positive polymerase chain reaction or MP serology of ≥1:160) was the MP group; a history of definite or suspected use of common sensitizing drugs (e.g., antibiotics, nonsteroidal anti-inflammatory drugs (NSAIDs), anticonvulsants, and proprietary pharmaceutical preparations) prior to onset of illness as derived by the ALDEN algorithm was the drug group. The study was approved by the Institutional Ethics Committee of Hebei Children’s Hospital (No.:202027; date: February 12, 2020), and written informed consent was obtained from the guardians of the participants.

### Inclusion criteria:

- Meeting the Bastuji-Garin diagnostic criteria.^8^
- Aged 1-12 years old.
- Typical rash, target-like erythema, diffusing and fusing into patches, rapidly progressing to flaccid blisters with epidermal exfoliation.
- Involvement of two or more mucosal tissues.
- Positive Nikolsky’s sign.
- Skin biopsy of atypical rash suggests necrosis of the whole epidermis.
- Stevens-Johnson syndrome is defined as epidermal exfoliation area <10% of the body surface area (BSA), TEN is the ratio of exfoliation area to BSA >30%, and the overlap of SJS/TEN is defined as the ratio of BSA 10%-30%.

### Exclusion criteria:

- Autoimmune bullous diseases.
- Staphylococcal scalded skin syndrome.
- Other types of severe drug rash.
- Other clear pathogenic infections.

The inpatient records of all children in both groups were retrospectively analyzed, from which gender, age, season of onset, history of out-of-hospital medication, time from onset to admission, clinical manifestations, maximum epidermal detachment area (including blisters, partially or completely peeled skin and Nikolsky-positive skin, excluding unpeeled areas), mucosal involvement, and prognosis were collected.

### Auxiliary examinations:

The first white blood cell count (WBC), hemoglobin, platelet count (PLT), C-reactive protein (CRP), procalcitonin (PCT), MP polymerase chain reaction, MP serological results (particle agglutination, IgM and IgG mixing), blood gas analysis, blood glucose, liver and kidney function, electrolytes, and serum albumin were examined on admission, and the severity-of-illness score for toxic epidermal necrolysis (SCORTEN) was calculated on day 1-3 of admission.^9^

### Therapeutic measures:

Ventilator support, blood purification, application of systemic corticosteroids (Cs) and intravenous immunoglobulin (IVIG).

### Statistical analysis:

All data were statistically analyzed using the SPSS 24.0 software (SPSS Inc., Chicago, IL, USA). The sample size is estimated by 95% confidence interval. Normally distributed measurement data were expressed as mean ± standard deviation (*X̅±S*), and t-test was taken for comparison between two groups; non-normally distributed measurement data were expressed as median and interquartile spacing [(M) (P25, P75)], and the Mann-Whitney U test was adopted for comparison between the two groups. Enumeration data were expressed as frequency (percentage) [n (%)], and comparisons between the two groups were tested by the chi-square test or Fisher’s exact method.

## RESULTS

The median age at onset of disease in the MP group was 108.0 (54.0, 129.0) months, which was greater than 42.0 (22.5, 75.0) months in the drug group, with a statistically significant difference (*P*<0.05); while the MP group was similar to the drug group in gender distribution, fever, cough, skin mucous membrane involvement and SJS/TEN distribution, with no statistically significant difference (*P*>0.05).

A total of 22 cases were included in the drug group, all of them had a clear or suspected history of drug use before the onset of the disease, with common sensitizing drugs were NSAIDs (12, 54.5%) and Chinese patent medicines (CPMs) (12, 54.5%), followed by antibiotics (9, 40.9%), and anticonvulsants (2, 9.1%). NSAIDs were the most common with amidopyrine (8, 36.4%), CPMs included Tanreqing Injection, Reduning Injection, QingKaiLing, and Potassium Sodium Dehydroandrographolide Succinate, while the antibiotic group was characterized by Amoxicillin and Clavulanate Potassium as the main causative agent (6, 27.3%). Drugs were taken orally in five cases (oxcarbazepine in two cases, penicillamine in one case, pyrethroids in one case, fluconazole in one case), and intramuscular injections or intravenous preparations were used in four cases (NSAIDs in two cases, and CPMs in two cases). The latency time from administration to symptom onset varied widely among drugs: around four weeks for oxcarbazepine and penicillamine, two weeks for fluconazole, and mostly 2-5 days for the rest. A single drug was taken in nine cases, and two or more drugs were used before the occurrence of SJS and TEN in 13 cases, and all of them were administered by a mixture of oral and intramuscular injections. (Table-I).

**Table-I:** Baseline characteristics, laboratory tests, and comorbidities in children with SJS/TEN in the MP and drug groups.

Item	MP-related group (n=20)	Drug-related group (n=22)	Statistic	P
Male [n (%)]	10(50)	10(45.5)	c^2^=0.087	0.768
Monthly age [M(P_25_~P_75_)] score	108.0(54.0, 129.0)	42.0(22.5, 75.0)	Z=-2.458	0.014
Onset to hospitalization [M(P_25_~P_75_)] days	6.8(3.3, 10.0)	5.4(3.0, 7.0)	Z=-0.951	0.342
SJS [n (%)]	15(75.0)	12(54.5)	c^2^=1.909	0.167
SJS/TEN [n (%)]	3(15.0)	5(22.7)	——	0.414
TEN [n (%)]	2(10.0)	5(22.7)	——	0.700
Fever [n (%)]	19(95.0)	21(95.5)	c^2^=0.000	1.000
Cough [n (%)]	14(70.0)	9(40.9)	c^2^=1.739	0.187
Oral and labial mucosal involvement [n (%)]	20(100)	21(95.5)	c^2^=0.000	1.000
Ocular conjunctival involvement [n (%)]	16(80.0)	19(86.4)	c^2^=0.019	0.890
Vulvar perianal mucosal involvement [n (%)]	12(60)	15(68.2)	c^2^=0.305	0.580
Sensitizing drugs [n (%)]				
Antibiotics	——	9(40.9)	——	——
Chinese patent medicines	——	12(54.5)	——	——
Anticonvulsants	——	1(4.5)	——	——
NSAIDs	——	12(54.5)	——	——
Other drugs	——	2((9.1))	——	——
≥2 drugs	——	13(59.1)	——	——
MP [n(%)]				
Antibody positive	19(95.0)	——	——	——
PCR positive	13(65.0)	——	——	——
WBC (×10^9^/L)	10.43±4.38	9.97±5.65	t=0.291	0.514
HGB (g/L)	119.8±17.9	120.4±14.9	t=-0.120	0.439
PLT (×10^9^/L)	319.9±136.4	266.0±112.8	t=1.402	0.588
E (×10^9^/L)	0.09(0.05, 0.32)	0.26(0.02, 0.59)	Z=-0.960	0.337
CRP (mg/L)	23.5(11.3, 41.8)	9.6(3.1, 36.6)	Z=-1.612	0.107
PCT (μg/L)	0.66(0.11, 1.99)	0.49(0.15, 2.00)	Z=-0.214	0.830
Neutrophils/lymphocytes	3.9±1.0	3.4±1.1	t=1.378	0.176
Complications [n(%)]				
Sepsis	1(5.0)	5(22.7)	——	0.187
Acute liver injury	6(30.0)	8(36.4)	c^2^=0.191	0.662
Consolidation of lung/pleural effusion	10(50.0)	2(9.1)	c^2^=8.591	0.003
Gastrointestinal bleeding	1(5.0)	2(9.1)	——	1.000
Hypoproteinemia	4(20.0)	4(18.2)	——	1.000
Occlusive fine bronchitis	2	0	——	0.221
Length of hospitalization [M(P_25_~P_75_)]days	14.5(11.0, 28.8)	14.5(9.0, 20.0)	Z=-1.112	0.266
Death	0	3	——	0.233

All 42 cases were treated with Cs. In addition to azithromycin for anti-infection, 6 (30.0%) cases in MP group received Cs shock therapy (methylprednisolone 20 kg/mg/d for three days and then tapered off), and 14 (65.0%) cases received conventional Cs dose (methylprednisolone 2kg/mg/d), 7 (35%) cases received IVIG (1g/kg/d for two days), and 5 (25.0%) cases received combined therapy of Cs shock plus IVIG. In the drug group, 15 (68.2%) cases were given Cs shock, 18 (81.8%) received IVIG, and 14 (63.2%) cases received Cs shock combined with IVIG. The proportion of children receiving Cs shock combined with IVIG in the MP group was significantly lower than that in the drug group, with a statistically significant difference (*P*<0.05). When SCORTEN was two points, the proportion of the MP group receiving IVIG application was significantly lower in the drug group, and the difference was statistically significant (P=0.011). As the SCORTEN score increased, the proportion of Cs shock and IVIG application increased in both groups. The relationship between therapeutic regimen and SCORTEN score is shown in the following Table-II.

**Table-II:** SCORTEN scores and treatment in children with SJS/TEN in the MP and drug groups.

Group	0-1			2			3			4		
	Cases	Cs shock	IVIG	Cases	Cs shock	IVIG	Cases	Cs shock	IVIG	Cases	Cs shock	IVIG
MP-related group	9	1(11.1)	0	6	2(33.3)	2(33.3)	1	0	1(100)	4	3(75.0)	4(100)
Drug-related group	5	2(40.0)	1(20.0)	9	5(55.6)	9(100)	4	4(100)	4(100)	4	4(100)	4(100)
P		0.505	0.357		0.608	0.011		0.200	1.000		1.000	1.000

The expected morbidity and mortality rate predicted by SCORTEN was calculated using the following formula: p (death) = elogit /(1 + elogit), with logit = -4.448 + 1.237 (SCORTEN).^9^ In the MP group, there were seven cases with a score of 0 on the first day of admission, two cases with a score of one, six cases with a score of two, one case with a score of three, and four cases with a score of 4. In the drug group, there were five cases with a score of 0 on the first day of admission, 0 cases with a score of one, nine cases with a score of two, four cases with a score of 3, and four cases with a score of 4. The median SCORTEN scores of the two groups were 2 (0, 2.8) and 2 (1.5, 3.0), respectively, with no statistically significant difference between the groups (*P*=0.388). The predicted mortality corresponding to SCORTEN score two was 12.2%. In this study, there were 0 actual deaths in the MP group, whereas there were three actual deaths (13.6%) in the drug group, including one death from congenital ventricular septal defect, combined with pulmonary hypertension, one death from acute respiratory distress syndrome and multi-organ failure, and one death from sepsis in the presence of severe malnutrition.

## DISCUSSION

A total of 67 children with SJS and TEN were admitted during the study period. Infection (34, 50.7%) was the most common cause, especially MP infection, followed by drugs (22, 32.8%), NSAIDs and CPMs were the most common causes. In contrast, adult SJS and TEN were mainly caused by drugs,^10^ with the distribution of common sensitizing drugs also different from that of children. Children are susceptible to a variety of pathogenic microorganisms due to the immaturity of their immune system. After infection, cytotoxic CD8+ T cells and natural killer cells are activated, which in turn activate other immune cells to produce pro-inflammatory molecules and down-regulate Treg cells. Imbalance in the secretion of proinflammatory and anti-inflammatory factors causes further amplification of the cascade response and secretion of granulysin, which induces apoptosis of keratinocytes due to the interaction of Fas ligand and Fas apoptosis receptor on the surface of keratinocytes,^11^ thereby triggering SJS and TEN. The injectable CPMs has emerged as a new type of sensitizing drug and replaced antiepileptic drugs, the reason may be that the skin necrosis induced by the carbamazepine/oxamazepineis clearly associated with human leukocyte antigen(HLA) typing in Asian populations,^12^ and the detection of HLA alleles avoids genetic susceptibility to SJS and TEN. TCM injections can induce sensitization by directly or indirectly stimulating mast cell degranulation release of histamine and activation of the complement system.^13^

The median age of onset was nine years in the MP group, which was higher than 3.5 years in the drug group, with a statistically significant difference(*P*<0.05), which may correlate with the epidemiology of mycoplasma pneumoniae infection. One large-scale study on CAP in children in the United States^14^ suggest that age is a major factor influencing the distribution of mycoplasma pneumoniae, mostly seen in school-age children. Little difference was observed between the two groups in terms of blood counts, immunologic function, and liver function. The MP group, in contrast to the drug group, had more cases with consolidation of lung and pleural effusion, with a higher need for ventilator support, and may be left with respiratory complications, especially with bronchiolitis obliteras.^15^

Despite reports of successful treatment with cyclosporine, TNF-a antagonists, and recombinant human type II tumor necrosis factor receptor-antibody fusion proteins^16^ in children with SJS and TEN, the guidelines still consider that there is no standardized drug intervention regimen.^17^ Most pediatric institutions in China still regard Cs and IVIG as the first-line treatment options. In this study, the proportion of MP group receiving Cs shock therapy and IVIG was lower than that of the drug group, but with no deaths, suggesting a good prognosis for the MP group. This may be associated with the pathogenesis, effective anti-infection and immune intervention.

In this study, positive MP-PCR was detected in the epidermal blister fluid of three children, indicating the existence of a direct mechanism of skin destruction by MP. All children in the MP group were treated with macrolide antibiotics, which blocked the action of transpeptidase, interfered with mRNA displacement, and selectively inhibited the synthesis of MP protein. This resulted in timely and effective removal of pathogens, reducing direct skin damage and limiting disease duration and severity; this is consistent with the findings of Wang et al.^18^

After MP infection, the proliferation of polyclonal B cells and antibody production are induced by molecular simulation mechanism, which leads to immune complex deposition and complement activation; it also induces an imbalance in T lymphocyte subsets, with a decline in helper T lymphocytes and a rise in cytotoxic T lymphocytes, generating an overactive inflammatory response that induces skin damage.^19^ Conventional doses of Cs suppress the immune response by reducing the proliferation of circulating cytotoxic T-lymphocytes and B-cells while inhibiting the function of monocytes/macrophages and down-regulating inflammatory factors and complement activation. In the drug group, a delayed hypersensitivity reaction (type IV) with Fas ligand and granulysin, mainly via the perforin/granzyme pathway, is observed.^20^ IVIG blocks FasL production and keratinocyte apoptosis. The actual mortality rate of MP group was significantly lower than predicted, suggesting that the SCORTEN score has a reduced ability to predict mortality risk in MP group, but it has guiding significance for treatment.

### Limitations:

First of all, polymerase chain reaction or serologic total antibody titer is applied as a test for MP in this study, which cannot effectively distinguish between previous and present infections. Therefore, the time interval relationship between MP infection and the occurrence of SJS/TEN cannot be clear. Second, this study is a single-center retrospective study with a long time span, small sample size, and missing immunological indicators in some children, which fails to compare immune function and cytokine levels between the two groups, limiting the breadth and depth of analysis. In response, more samples need to be included in follow-up studies, and follow-up time should be extended to further evaluate comparison of treatment efficacy and prognosis.

## CONCLUSIONS

In conclusion, MP infection and drugs constitute the predominant triggers of SJS/TEN in children. The MP group is mostly seen in school-aged children who tend to have consolidation of lung/pleural effusion and high need for assisted ventilation compared to the drug group; however, the group shows a good prognosis with a decreased predictive ability of SCORTEN for their mortality risk. For those with SCORTEN≦2 points, macrolides combined with conventional dose Cs may be the first-line treatment option for them. And for those with SCORTEN score ≥3 points, it may be a node for the MP group to apply Cs shock and or IVIG treatment.

### Authors’ Contributions:

**XW, FG:** Carried out the studies, participated in collecting data, and drafted the manuscript and are responsible and accountable for the accuracy or integrity of the work.

**LK**, **YJ** and **LJ:** Performed the statistical analysis and participated in its design. Critical Review.

All authors have read and approved the final manuscript..
